# Advanced Glycation End Products in Disease Development and Potential Interventions

**DOI:** 10.3390/antiox14040492

**Published:** 2025-04-18

**Authors:** Yihan Zhang, Zhen Zhang, Chuyue Tu, Xu Chen, Ruikun He

**Affiliations:** 1 School of Biology and Biological Engineering, South China University of Technology, Guangzhou Higher Education Mega Center, Panyu District, Guangzhou 510006, China; zhangyh7@by-health.com (Y.Z.); zhangz10@by-health.com (Z.Z.); 2 BYHEALTH Institute of Nutrition & Health, No. 916, Huangpu Avenue East, Huangpu District, Guangzhou 510799, China; tucy@by-health.com (C.T.); chenx8@by-health.com (X.C.)

**Keywords:** advanced glycation end products, chronic diseases, intervention, natural products

## Abstract

Advanced glycation end products (AGEs) are a group of compounds formed through non-enzymatic reactions between reducing sugars and proteins, lipids, or nucleic acids. AGEs can be generated in the body or introduced through dietary sources and smoking. Recent clinical and animal studies have highlighted the significant role of AGEs in various health conditions. These compounds accumulate in nearly all mammalian tissues and are associated with a range of diseases, including diabetes and its complications, cardiovascular disease, and neurodegeneration. This review summarizes the major diseases linked to AGE accumulation, presenting both clinical and experimental evidence. The pathologies induced by AGEs share common mechanisms across different organs, primarily involving oxidative stress, chronic inflammation, and direct protein cross-linking. Interventions targeting AGE-related diseases focus on inhibiting AGE formation using synthetic or natural antioxidants, as well as reducing dietary AGE intake through lifestyle modifications. AGEs are recognized as significant risk factors that impact health and accelerate aging, particularly in individuals with hyperglycemia. Monitoring AGE level and implementing nutritional interventions can help maintain overall health and reduce the risk of AGE-related complications.

## 1. Introduction

Advanced glycation end products (AGEs) are harmful compounds formed through non-enzymatic reactions involving reducing sugars and proteins [1]. Their discovery dates back to 1912, when non-enzymatic browning was observed in foods and beverages during the heating of glucose with glycine [2]. This reaction enhances the flavors and aromas of foods rich in sugar and protein.

However, AGEs have detrimental effects on tissues and organs. They can directly cross-link proteins, altering their structure and function, or indirectly contribute to disease by generating reactive oxygen species (ROS) and pro-inflammatory cytokines [3]. AGEs are naturally produced in the body as part of normal metabolism but can also form during food cooking and processing. Once ingested, they are absorbed through the gastrointestinal tract and may accumulate in excess [4]. Accumulated AGEs bind to their receptors (RAGEs), promoting oxidative stress and disrupting intracellular signaling. This contributes to the progression of chronic diseases, including diabetes [5], cardiovascular disease [6], cognitive impairment [7], and aging-related conditions [8], as well as disorders affecting the liver [9], lungs [10], eyes [11], intestines [12], and reproductive system [13]. Therapeutic strategies aimed at mitigating the impact of AGEs include inhibiting their formation, enhancing their clearance, breaking down established AGE-related protein cross-links, and blocking their interaction with RAGE.

This review examines recent advancements in AGE research, highlighting their role in various diseases and underlying pathological mechanisms, with a particular focus on clinical evidence. Additionally, it highlights recent studies identifying small-molecule inhibitors and natural compounds that target AGEs, offering insights into reducing AGE-related harm through nutritional interventions.

## 2. Advanced Glycation End Product (AGE) Introduction and Classification

### 2.1. AGE Formation

**Figure 1:**
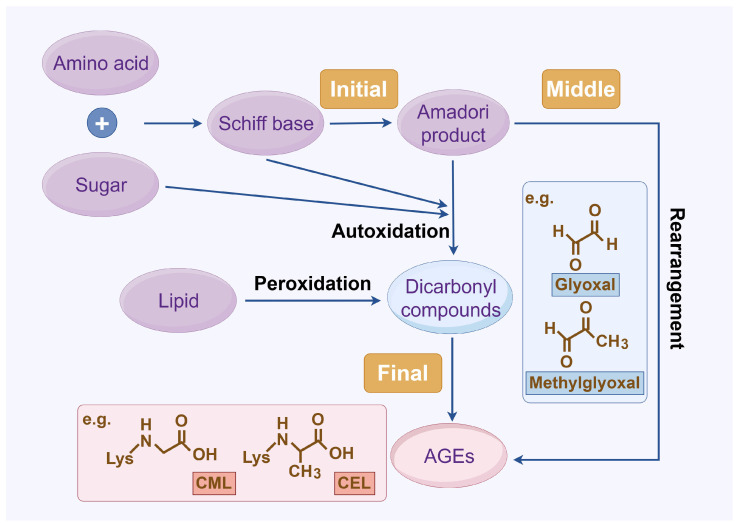
AGE formation. Abbreviations: AGEs, advanced glycation end products; CEL, carboxy ethyl lysine; CML, carboxyl methyl lysine.

The complex series of reactions that contribute to AGE formation is collectively known as the Maillard reaction. In this process, the terminal amino groups of amino acids, as well as the side-chain amino groups of lysine and arginine residues in proteins, react non-enzymatically with the carbonyl groups of reducing sugars (e.g., glucose, ribose, and trioses) to form unstable Schiff bases (early AGEs). Under oxidative stress, these Schiff bases undergo rearrangement or glycoxidation, leading to the formation of final AGEs or highly reactive 1,2-dicarbonyl compounds, such as glyoxal and methylglyoxal. These dicarbonyl compounds, which also arise from glucose autoxidation, lipid peroxidation, and the polyol pathway, react with protein amino groups, leading to both intra- and inter-protein cross-linking, and then they undergo oxidation, dehydration, or polymerization [14]. The resulting stable protein modifications, along with the small molecular products formed from their degradation, are collectively known as AGEs, with carboxymethyl lysine (CML) and carboxyethyl lysine (CEL) being the most widely studied (Figure 1) [15]. While this process occurs naturally over time, it is promoted by hyperglycemia, oxidative stress, and metabolic imbalances.

### 2.2. AGE Type

**Figure 2:**
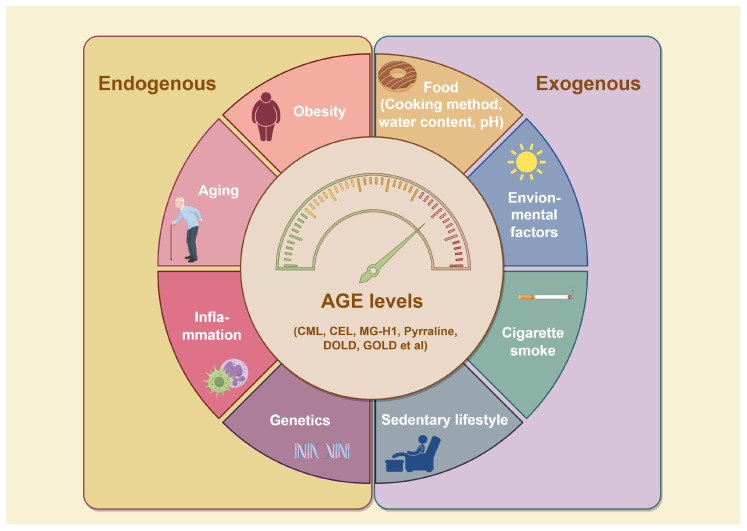
Factors influencing AGE formation. Reducing sugars and amino compounds form Schiff bases through non-enzymatic reactions, which are subsequently converted into Amadori products through reduction and rearrangement. These Amadori products further degrade into highly reactive dicarbonyl intermediates, which react with amino groups in proteins and other biomolecules like lipids, leading to the formation of stable AGEs. AGE accumulation is influenced by both internal and external factors. Internally, genetic factors, inflammation, aging, and metabolic disorders like obesity accelerate AGE formation. Externally, factors such as a sedentary lifestyle, smoking, UV exposure, and consumption of AGE-rich foods further contribute to AGE buildup. Abbreviations: AGEs, advanced glycation end products; CEL, carboxy ethyl lysine; CML, carboxyl methyl lysine; DOLD, 1,3-di(Nε-lysino)-4-(2,3,4-trihy -droxybutyl)-imidazolium; GOLD, 6-{1-[(5S)-5-ammonio-6-oxido-6-oxohexyl] imidazolium-3-yl} -L-norleucine; MG-H1, Methylglyoxal-derived hydroimidazolone-1.

To date, over 20 distinct types of AGEs have been identified in the human body and dietary sources, each exhibiting a range of properties and chemical structures [15]. AGEs can form endogenously, either intracellularly or extracellularly, or be acquired exogenously, primarily from food and tobacco (Figure 2) [3]. The endogenous production of AGEs is influenced by carbohydrate precursor availability, ROS levels, and protein turnover rates [16]. Representative products include pentosidine, CML, and methylglyoxal (MGO), which can serve as markers of pathophysiological processes [17]. While exogenous AGEs are structurally and functionally identical to endogenous ones, they play a critical role in the accumulation and systemic circulation of AGEs [3]. Consequently, exogenous AGEs, primarily from dietary sources, are regarded as the predominant contributors to the body’s overall AGE burden [18]. The formation of AGEs in food primarily depends on factors such as cooking temperature, water content, pH, cooking time, and cooking method. Additionally, cigarette smoke contains highly reactive glycation products [8]. Together, systemic AGE concentrations are determined by a combination of dietary intake, genetics, age, and overall health status. Elevated AGEs can synergistically trigger inflammation and oxidative stress, resulting in various adverse health outcomes.

## 3. AGE-Related Diseases

### 3.1. Mechanisms of AGE-Induced Pathology

AGEs accumulate in almost all mammalian tissues and cause chronic pathology [19]. The primary mechanisms of AGE-induced pathology include direct protein trapping and cross-linking, which alter protein structure and function, as well as indirect activation of intracellular signaling through both receptor- and non-receptor-mediated pathways. These processes lead to increased production of inflammatory cytokines and ROS [3,5].

Representatively, covalent cross-linking of AGEs to collagen or elastin in the extracellular matrix (ECM) slows the hydrolytic breakdown of these proteins and reduces their flexibility. This process contributes to the thickening of the basement membrane, compromising the integrity of blood vessels and increasing the risk of hemorrhagic pathologies [20]. Additionally, AGE-induced skin aging is also governed by the simple act of covalently cross-linking to collagen and elastin, especially types I and IV collagen [21]. This process causes a loss of skin elasticity and hydration. Moreover, the accumulation of AGEs in skin tissue also leads to skin yellowness [22].

Besides direct trapping or cross-linking the proteins, AGEs also act by modifying proteins or forming adducts with them through binding with AGE ligand-gated receptors, such as the most well-known RAGE and its secreted soluble isoform, sRAGE [23]. RAGE, encoded by a gene on chromosome 6, is a transmembrane protein belonging to the immunoglobulin superfamily of cell surface receptors. It is distributed across various organs but is most enriched in the lung, heart, and skeletal muscle. RAGE is expressed at basal levels under normal conditions but is significantly elevated under pathological conditions or chronic inflammation [4]. The activation of RAGE induces a cascade of inflammatory reactions that start with the activation of transcription factor NF-κB. Then, it triggers a variety of downstream effectors, including mitogen-activated protein kinase (MAPK), p38, Ras-mediated extracellular signal-regulated kinase (ERK1/2), Janus kinase signal transducer and activator of transcription (JAK/STAT), and the stress-activated protein kinase/c-Jun N-terminal kinase (SAPK/JNK) pathway, that in turn will lead to sustained activation of transcription factors such as NF-κB, HIF-1α, STAT3, and AP-1 [5]. These signaling pathways regulate various physiological processes, triggering proliferative, angiogenic, fibrotic, thrombogenic, and apoptotic responses, ultimately contributing to AGE-induced pathology in various tissues [24,25].

The AGE–RAGE interaction could also increase the ROS levels through the activation of mitochondrial and NADPH (nicotinamide adenine dinucleotide phosphate oxidase) pathways [26], which in turn leads to increased production of AGEs and a vicious cycle of intracellular damage [27].

The ROS is accumulated from the conversion of glucose to fructose through the NADPH pathway. The decreased endogenous antioxidant mechanisms, such as the depletion of GSH, increase the concentrations of glyoxal (GO) and MGO, thereby promoting the glycation process [28].

The soluble forms of AGEs, sRAGE, lacking the transmembrane and cytoplasmic domains, are distributed through the circulation [29]. The extracellular domain of RAGE released from the cells to the circulation is called endogenous secretory RAGE (esRAGE) [30]. It is reported that the esRAGE may act as a decoy, which cancels the effects of AGEs in cultured cells and reverses diabetic vascular dysfunction in mice [31]. The RAGE and esRAGE are suggested to be involved in feedback regulation of the toxic effects of RAGE-mediated signaling.

Therefore, more and more studies have been conducted to explore the role of sRAGE and esRAGE in various pathophysiologic conditions, and it has been found that they could potentially serve as promising prognostic markers. However, according to some research, the relationship between sRAGE and cardiovascular diseases is inconsistent, suggesting further studies are needed [32,33,34,35].

### 3.2. AGEs and Diabetes Mellitus

Diabetes mellitus refers to a group of chronic metabolic disorders characterized by elevated blood glucose levels (hyperglycemia) resulting from defects in insulin secretion, insulin action, or both [36,37]. Persistent hyperglycemia leads to both microvascular and macrovascular complications, making diabetes a major public health concern. One of the harmful consequences of hyperglycemia is the accelerated formation and accumulation of AGEs, which play a key role in the development of nearly all diabetic complications [38]. However, emerging research suggests that AGEs may contribute to the onset of diabetes rather than simply being a consequence of it. In healthy individuals, dietary intake of AGE-rich foods is the primary source of these compounds. AGEs act as pro-oxidants and appetite stimulants, contributing to overnutrition, inflammation, obesity, and ultimately, diabetes mellitus [39].

Although numerous studies have linked AGEs to the occurrence and severity of diabetes-related complications, few cohort studies have specifically investigated the relationship between serum AGE levels and the onset of diabetes itself. According to a stratified case-cohort analysis of the Atherosclerosis Risk in Communities (ARIC) study, 543 middle-aged individuals who developed diabetes and 514 individuals who did not were followed over a median of 9 years. The study found that elevated levels of CML were associated with an increased risk of diabetes, particularly among those with impaired fasting glucose and among white participants. This suggests that elevated fasting CML may predict the development of incident diabetes [40]. Moreover, as consistently demonstrated by a large hospital-based case-control study and a nested case-control study within an ongoing community-based prospective cohort of Chinese adults, higher plasma AGEs and lower plasma sRAGE were significantly associated with increased risk of Type 2 diabetes (T2D) among adults without cardiovascular diseases. Specifically, positive associations were observed between plasma CML, CEL, and methyl glyoxal-derived hydroimidazolone 1 (MG-H1) levels and T2D, after adjusting for other factors [41]. Other studies have found that individuals of all ages who consume a diet high in AGEs may have elevated levels of high-sensitivity C-reactive protein (hs-CRP), tumor necrosis factor (TNF), fibrinogen, vascular cell adhesion molecule 1 (VCAM-1), and homeostatic model assessment (HOMA). These findings only indirectly suggest that AGEs may contribute to the development of diabetes. In animal studies, the evidence linking AGEs to diabetes is more direct. The nonobese diabetic (NOD) mouse, as a model of autoimmune type 1 diabetes mellitus (T1DM), usually becomes diabetic at the age of 4–5 months. However, an AGE-restricted diet could significantly reduce the incidence of T1DM in these NOD mice, even to less than 15% in the next two generations, as long as the AGE-restricted diet (the semipurified standard diet, AIN-93G, prepared without the second step of heating, containing fivefold less CML-like AGE and 4.5-fold less MG-derived epitopes compared to the identical chow mix processed under the standard procedure) was maintained in both dams and offspring [42]. Similar results were observed in type 2 diabetes mellitus (T2DM) model mice, when an AGE-restricted diet (produced with less exposure to heat and containing less CML, MG, or total AGEs measured by ELISA) was administered [43,44,45]. Several animal experiments have shown that simply reducing basal oxidative stress by dietary AGE restriction could sharply reduce the risk of diabetes mellitus and its complications [39].

In individuals with diabetes, AGE levels are strongly associated with disease severity and the extent of complications affecting various organs and tissues. This relationship will be explored in detail later in this review.

The pathophysiology of diabetes mellitus induced by AGEs primarily involves direct protein trapping and cross-linking, or indirect binding to cell surface receptors, with the RAGE being the most significant [46,47]. As reported, the elevated AGEs in diabetic individuals could interact with RAGE and trigger various downstream pathways, including SAPK/JNK, MAPK, p38, ERK1/2, JAK/STAT, and PKC, causing sustained activation of NF-κB, AP-1, CREB, and STAT3. This would create a vicious cycle, involving inflammation, ROS generation, mitochondrial dysfunction, beta cell impairment, and apoptosis, all of which contribute to the pathology of insulin resistance [5]. Furthermore, Islet amyloid polypeptide (IAPP), which is found to be heavily glycated and to form toxic amyloid-like aggregates, is another major factor contributing to beta cell death [48]. It could be selectively bound by RAGE, leading to NADPH oxidase-mediated ROS generation, inducing cellular stress, inflammation, islet amyloidosis, and finally decreased beta cell mass [49].

### 3.3. AGEs and Cardiovascular Diseases

Cardiovascular diseases (CVDs) are defined as life-threatening chronic conditions affecting the heart and vascular system and are categorized as microvascular and macrovascular diseases. Microvascular disease leads to nephropathy, retinopathy, and neuropathy; macrovascular disease is usually caused by atherosclerosis, which leads to the narrowing or occlusion of blood vessels, including coronary artery disease, cerebrovascular disease, and peripheral artery disease [50].

The accumulation of AGEs (such as CML and 3-deoxyglucosone hydroimidazolone) is implicated in the onset and progression of cardiovascular complications in diabetic patients [6,51]. Serum AGE levels were higher in T2DM patients with either macrovascular or microvascular disease compared to those without complications [52]. As a pathologic basis for macrovascular disease, the progression and severity of atherosclerosis in diabetic patients have been reported to correlate with serum levels of AGEs [53]. In non-diabetic subjects with coronary heart disease, elevated AGE levels were associated with the number of vascular stenoses [54]. Additionally, tissue AGEs (skin autofluorescence) could serve as a predictor for diabetes-related heart failure and CVD mortality [55]. However, there is an inconsistency in the findings regarding the relationship between circulating sRAGE and CVDs. Some studies have found a negative correlation between atherosclerosis and circulating sRAGE, suggesting that sRAGE may be a protective factor against atherosclerosis [32,33]. However, other studies have shown a positive correlation between atherosclerosis and sRAGE [34,35]. The reason for the conflicting findings may be the differences in clinical study design and the strong correlation between serum sRAGE and other patient variables, such as age and diabetic background. As one of the common microvascular complications of diabetes, diabetic neuropathy could cause a range of symptoms, including foot ulcers [56]. Glycation has been reported to be an important pathophysiologic pathway leading to complications in foot ulcers [56]. The relevance of AGEs to diabetes-related nephropathy and retinopathy will be discussed in subsequent paragraphs.

In the circulatory system, the constituents of lipoproteins are particularly susceptible to advanced glycation, and glycated low-density lipoproteins (LDLs) may not be effectively recognized by their receptors [57,58]. These glycated particles can reach the arterial wall and be taken up by macrophage receptors, leading to lipid accumulation [57,58]. In addition, AGE–RAGE enhances the adhesion of macrophages and T-lymphocytes by upregulating adhesion proteins via the NF-κB signaling pathway [59]. This adhesion process induced continuous inflammatory responses, promoting plaque formation [60,61]. Furthermore, the AGE–RAGE axis regulates the expression of ATP-binding cassette transporter G1 (ABCG1) and cluster of differentiation 36 (CD36), facilitating the transformation of macrophages into foam cells [62,63]. The activation of transforming growth factor-β (TGF-β) by AGE–RAGE stimulated the proliferation and migration of arterial smooth muscle cells [64], which further disrupted the endothelial cell barrier and promoted plaque formation in response to inflammation [65]. Activation of AGE–RAGE could inhibit the expression of endothelial nitric oxide synthase (eNOS), thereby reducing the production, release, and bioactivity of nitric oxide (NO) [66]. This inhibition led to endothelial dysfunction and impaired vascular contraction and dilation [66].

Numerous studies have highlighted the role of the AGE–RAGE axis in cardiac dysfunction. Prolonged elevation of blood glucose levels led to increased non-enzymatic glycation of proteins involved in calcium (Ca^2+^) homeostasis, including sarcoplasmic reticulum Ca^2+^-ATPase (SERCA) and ryanodine receptors (RyR) [67,68]. The cross-linking of SERCA and RyR induced by AGEs disrupts Ca^2+^ handling, adversely affecting myocardial relaxation and contraction, which ultimately results in diastolic dysfunction [67,68].

### 3.4. AGEs and Chronic Kidney Diseases

As reported in many studies, chronic kidney disease (CKD) is marked by excessive production and accumulation of AGEs, which in turn promote CKD-related morbidities and mortality [69]. Urinary uromodulin (UMOD) is glycated in diabetic kidney disease (DKD) and forms AGEs, and glcUMOD may serve as a biomarker for DKD [70]. The free adduct of MG-H1 serves as a risk marker for CKD in both non-diabetic and diabetic patients [71]. Moreover, in poorly controlled diabetic patients, persistently low serum MG-H1 levels correlate with a reduced incidence of DKD [72]. Some early-type glycation products, as the precursors of AGEs, cause vascular damage through oxidative stress and have a close clinical association with CKD. The higher MGO concentrations were demonstrated to be associated with CKD and may be useful in determining the prognosis of the disease [73]. A recent prospective cohort study demonstrated that the glycated albumin (GA) level in CKD patients, an early Amadori-type glycation protein and a precursor to harmful AGEs, was independently associated with risks of end-stage kidney disease, CVDs, and mortality, regardless of diabetes status [74]. The AGEs could be evaluated using skin autofluorescence, and a recent study suggested the fluorophore AGE-to-NADH ratio to be a new biomarker for the presence of diabetic CKD [75]. In addition, elevated circulating esRAGE is independently associated with CKD and is an independent predictor of incident CKD in older community-dwelling adults [76]. The sRAGE was also reported to be associated with CKD incidence [77], and AGE/sRAGE can be an additional risk factor for CKD progression over a longer time [78].

The AGEs contribute to the pathology of CKD and CVD mainly as pro-oxidative and pro-inflammatory factors [79]. AGEs increase the levels of ROS through activation of NADPH oxidase and mitochondrial pathways in both a receptor-dependent and independent manner. Simultaneously, AGEs amplify inflammatory responses in patients with CKD through the AGE–RAGE axis, most significantly by activating the NF-kB. Additionally, several studies demonstrated that the expression of some Nrf2 target antioxidant genes is decreased in animal models and patients of CKD, which may consequently aggravate oxidative stress and inflammation [79]. Furthermore, AGE-induced direct modification and cross-linking of the proteins cause numerous structural changes, including alterations in surface charges, packing density, and structural integrity, leading to stiffening of the vasculature as well as the expansion of cellular basement membranes in diabetes and CKD [80].

### 3.5. AGEs in Joint Diseases

AGEs also accumulate in cartilage, bone, and synovium, particularly in individuals with type 2 diabetes, which disrupts normal cellular functions, alters collagen structure, promotes tissue deterioration, and leads to skeletal fragility [81]. Particularly, AGEs would cross-link collagen fibers in the cartilage, leading to stiffness and reduced elasticity. Studies in the Health, Aging, and Body Composition cohort showed that higher CML levels in T2D were associated with an increased risk of incident clinical fractures, independent of bone mineral density (BMD) and other factors. However, this association was not observed in non-diabetic individuals, and CML was not significantly linked to vertebral fractures in either group [82]. Pentosidine levels from urine are negatively associated with trabecular bone scores [83], and circulating pentosidine correlates with bone mechanical properties [84]. A meta-analysis involving 5878 participants reported that higher urinary pentosidine levels are linked to an increased fracture risk and, although less strongly, to lower bone mineral density, highlighting its potential as a biomarker of impaired bone health [85]. These findings suggest that AGEs, particularly CML and pentosidine, would contribute to the decreased resilience of cartilage and cause bone fragility in T2D, highlighting their role in the pathogenesis of bone-related complications in diabetic patients.

RAGE plays a pivotal role in the pathogenesis of joint diseases by mediating inflammatory responses and contributing to tissue damage, with high expression observed in joint tissues. Through their interaction with RAGE, AGEs can induce the expression of pro-inflammatory cytokines and osteoclastogenic markers in fibroblast-like synovial cells, leading to the structural and functional impairment of joints [86]. In rheumatoid arthritis (RA), higher expression of RAGE has been noted in synovial tissues, particularly in macrophages and endothelial cells, where it amplifies the inflammatory response by activating the NF-κB signaling pathway [87]. Additionally, CML was detected in both RA and osteoarthritis (OA) synovial tissues, where it may interact with RAGE on macrophages and T cells, potentially triggering autoimmune responses that worsen joint inflammation [88]. The prominent presence of RAGE in RA compared to OA suggests a more significant role in the inflammatory processes that characterize these conditions [89]. Moreover, soluble RAGE levels in plasma and synovial fluid are inversely related to the severity of knee osteoarthritis [90]. Together, these findings underscore the critical role of AGEs and RAGE in the propagation and exacerbation of joint diseases, highlighting their potential as therapeutic targets in arthritis.

Recent clinical studies have also confirmed the critical roles of AGEs and RAGE in the pathogenesis of joint diseases, especially in RA. AGEs and advanced oxidation protein products (AOPPs) were found to be significantly elevated in RA patients compared to healthy controls [91]. Additionally, the presence of anti-AGE antibodies was observed in nearly half of RA patients, while only 7.5% of healthy controls tested positive; they were associated with genetic risk factors, inflammation, and radiological progression, particularly in seronegative RA [92]. Moreover, the inhibition of AGEs with benfotiamine, pyridoxamine, and methylcobalamin has shown promising results in reducing endothelial dysfunction and inflammatory disease activity in RA, suggesting that AGEs contribute to the vascular and systemic inflammation characteristics of this condition [93]. Furthermore, low levels of sRAGE in female RA patients have been associated with increased cardiovascular risk, underscoring the need for metabolic health monitoring in these individuals [94]. Collectively, these findings suggest that targeting AGE formation or blocking RAGE signaling may present promising adjunctive strategies for managing arthritis, especially RA.

### 3.6. AGEs and Neurodegenerative Diseases

Neurodegenerative diseases (NDDs) encompass a diverse array of disorders characterized by irreversible neuronal damage and a progressive decline in neural function. These disorders include Alzheimer’s disease (AD), Parkinson’s disease (PD), amyotrophic lateral sclerosis (ALS), and prion diseases [95].

Over the past few decades, numerous studies have demonstrated a correlation between AGEs and brain dysfunction. The high oxygen consumption and glucose metabolism in the brain may contribute to the accumulation of MG-H1 and CEL [96]. AGEs have been detected in astrocytes exclusively in older individuals, including those with AD, but not in younger controls [97]. The number and proportion of AGE-positive neurons and astroglia are significantly elevated in AD patients [98,99]. Immunohistochemical studies show that AGEs accumulate notably in lipofuscin granules within the tissues of AD patients and the elderly [99]. Furthermore, increased levels of AGEs have been identified in Lewy bodies and toxic protein inclusions, which are recognized as pathological markers of PD [100], in neurons from PD patients [101]. In patients with ALS, AGEs levels, particularly CEL, CML, and pyrrole compounds, were significantly elevated in spinal cord astrocytes [102]. These findings indicate that the accumulation of AGEs in brain tissue is critical to the pathological progression of NDDs. Furthermore, increased levels of AGEs (such as CML and MG) have also been detected in the bodily fluids of patients with NGDs, including blood [103,104] and urine [105], alongside reduced levels of sRAGEs [106]. Elevated plasma AGEs have been associated with poorer cognitive function, with higher levels of CML correlating with a greater incidence of mild cognitive impairment [7]. These studies suggest that AGEs may serve as potential predictors for the onset and progression of NDDs.

The role of AGEs in NDDs is complex and multifaceted, involving several key mechanisms. Firstly, AGEs promoted the aggregation of amyloid-β peptides (Aβ) into insoluble fibrils, leading to the formation of amyloid plaques, a hallmark of AD pathology [107]. Additionally, the activation of glycogen synthase kinase-3 (GSK-3) by AGEs induced the excessive phosphorylation of Tau protein (p-Tau) [108]. In response to ROS disruption, Tau protein could interact with cyclin-dependent kinase 5 (CDK5) and GSK3β, leading to hyperphosphorylation [109]. Phosphorylated Tau specifically affected the activity of mitochondrial complex I, synergistically promoting Aβ-mediated mitochondrial dysfunction and ROS production [110]. These studies suggest that oxidative stress, mitochondrial disruption, and Aβ and p-Tau accumulation may form a vicious circle, and the causal relationship between them in AD needs to be further explored. The ROS production triggered by the AGE–RAGE axis could also amplify p-Tau and Aβ production, exacerbating their neurotoxicity [111]. Glycosylated α-synuclein and its oligomers were reported to induce toxicity in dopaminergic neurons in patients with PD [100]. Secondly, AGEs were involved in the disruption of the blood–brain barrier (BBB), which is primarily composed of endothelial cells, astrocytes, and pericytes [112]. Elevated AGE levels reduced the expression of integrin α1, platelet-derived growth factor receptor-1β (PDGF-R1β), and Connexin-43 (Cx-43), leading to pericyte dysfunction [113]. The upregulation of vascular endothelial growth factor (VEGF) and matrix metalloproteinase-2 (MMP-2) in pericytes was induced by AGE-activated autocrine TGF-β signaling, resulting in basement membrane hypertrophy of the BBB [114]. Additionally, high levels of AGEs decreased the expression of tight junction proteins, such as occludin and claudin, which compromises the integrity of tight junctions [115]. Thirdly, neuroinflammation resulting from the dysregulation of the AGE–RAGE axis is a significant factor in the progression of NDDs. The RAGEs could recruit the adaptor protein mDia1 upon dimerization, thereby activating various signaling pathways, including the JAK/STAT pathway [116]. The translocation of activated STAT to the nucleus promoted expression of various cytokine-responsive genes and activated the inflammatory response [117]. Furthermore, the AGE–RAGE pathway modulated the apoptosis of various cell types in the brain, including microvascular cells and neuronal cells [118,119]. The interaction of AGEs with RAGE could lead to the release of IL-8, causing dephosphorylation of the cytoplasmic nuclear factor of activated T cells (NF-AT) [120]. The increased expression of Fas ligand (FasL), induced by the translocation of NF-AT, could increase caspase activity and ultimately result in cell death [120].

### 3.7. AGEs and Skin Disorders

AGEs are key contributors to skin aging, especially in photoaging, a process exacerbated by sun exposure. Numerous studies have shown that AGEs accumulate more in sun-exposed areas than in protected skin, indicating that UV exposure accelerates their formation [121]. This buildup of AGEs leads to the cross-linking of collagen and elastin fibers, reducing skin elasticity and increasing stiffness, which results in visible signs of aging such as wrinkles, sagging, and other signs of photoaging [122]. Moreover, AGEs intensify the skin’s inflammatory response, hastening the degradation of extracellular matrix proteins such as collagen and elastin, further amplifying their harmful effects and worsening visible signs of aging such as wrinkles and the loss of firmness [123].

Research has also demonstrated that exposure to ultraviolet radiation A (UVA) in the presence of AGEs triggers the generation of ROS such as superoxide, hydrogen peroxide, and hydroxyl radicals. These ROS further escalate oxidative stress, damaging dermal fibroblasts, reducing cell viability, and causing additional cellular damage, all of which compromise the structural integrity of the skin [124]. In addition to structural damage, AGEs are implicated in hyperpigmentation disorders. In particular, they activate the NLRP3 inflammasome in fibroblasts, promoting the release of pro-inflammatory cytokines like IL-18, driving melanogenesis, and elevating melanin levels, which are linked to hyperpigmentation disorders such as melasma and solar lentigo [125]. Clinically, higher AGE levels correlate with increased trans-epidermal water loss (TEWL), reduced hydration, and increased melanin and erythema, all of which deteriorate skin quality [126]. Given their roles in oxidative stress, inflammation, and collagen cross-linking, AGEs are pivotal in skin aging and represent promising targets for anti-skin aging therapies, particularly for those with extensive sun exposure.

AGE formation is accelerated in hyperglycemic conditions, such as diabetes, where it significantly hinders wound healing [127]. AGEs promote oxidative stress and chronic inflammation, both of which are detrimental to effective tissue repair. In diabetic wounds, the AGE–RAGE axis triggers excessive ROS production and an exaggerated inflammatory response, which delays healing and increases tissue damage. Furthermore, AGEs can induce apoptosis in key cell types involved in wound repair, such as fibroblasts, macrophages, and endothelial cells, thereby disrupting normal wound closure processes [128]. In diabetic keratopathy, for example, AGEs hyperactivate the NLRP3 inflammasome, leading to delayed corneal wound healing and impaired nerve regeneration [129]. Similarly, in diabetic foot ulcers, AGEs inhibit macrophage function and increase apoptosis, further impairing the healing process [130]. Targeting the AGE–RAGE axis, either by blocking RAGE or reducing AGE formation, has shown promise in improving wound healing outcomes, highlighting AGEs as critical factors in diabetic wound pathogenesis [131,132].

AGEs also play significant roles in the pathogenesis of skin diseases such as psoriasis, where they are involved in both inflammation and metabolic comorbidities. Multiple studies highlight the accumulation of AGEs in the skin and blood of psoriasis patients, particularly in severe cases, where they are associated with disease severity and progression [133]. The AGE–RAGE axis amplifies inflammatory responses through the release of cytokines, chemokines, and ROS, further aggravating psoriatic lesions and promoting keratinocyte proliferation [134]. This interaction, mediated by the STAT1/3 signaling pathway, stimulates the production of pro-inflammatory cytokines like IL-36α, enhancing Th17 immune responses [134]. Additionally, polymorphisms in the RAGE gene have been associated with a higher risk of psoriasis, particularly in patients without common comorbidities like cardiovascular disease and diabetes [135]. In addition to their role in psoriasis, AGEs have been implicated in other skin disorders linked to metabolic dysfunction. For instance, AGEs play a role in the development of systemic sclerosis, a disease characterized by excessive collagen deposition and fibrosis [136]. Elevated AGE levels have been observed in the skin of scleroderma patients [137], and their interaction with RAGE is thought to exacerbate fibrosis by promoting collagen cross-linking and perpetuating chronic inflammation.

### 3.8. AGEs and Liver Diseases

Metabolic-associated fatty liver disease (MAFLD) affects about 25% of the global population and is commonly associated with CVDs such as hypertension and atherosclerosis [138]. Research has shown elevated serum glyceraldehyde (GA)-derived AGEs in MAFLD patients compared to healthy individuals [139]. These GA-derived AGE levels could potentially serve as biomarkers for distinguishing metabolic dysfunction-associated steatohepatitis (MASH) from simple steatosis. For instance, a study with 43 MASH patients treated with atorvastatin showed a significant decrease in serum GA-derived AGEs levels over 12 months [140]. Additionally, high serum AGE levels have been identified as independent risk factors for severe MAFLD-related steatosis [141]. Genetic studies further indicate that certain *RAGE* polymorphisms in obese patients double the risk of MASH [9]. Other studies reported increased AGE levels and reduced sRAGE in MAFLD patients, which correlates with an increased glycation/sRAGE ratio and an elevated risk of MAFLD [142,143,144]. Further findings show that serum CML levels were increased in MAFLD patients and correlated with markers of liver injury [145].

In alcoholic liver disease (ALD), a major cause of chronic liver issues, AGEs are thought to contribute to liver injury through the ‘toxic AGE theory’, wherein AGEs derived from acetaldehyde or glyceraldehyde interact with RAGE, activating inflammation and oxidative stress. Nearly all chronic heavy drinkers develop ALD, with 10–35% progressing to alcoholic steatohepatitis and 8% to 20% to cirrhosis [146]. Alcoholic cirrhosis is linked to 47.9% of all cirrhosis-related deaths [147]. Research has observed acetaldehyde-derived AGEs in liver tissues of individuals with alcoholism [148]. A particular AGE, AGE10, was suggested as a diagnostic marker for alcoholic steatohepatitis [149]. In addition, elevated RAGE expression in ALD patients correlates with levels of transferrin, hepcidin, and ferritin, underscoring the potential role of AGEs in ALD progression [150].

Regarding liver cancer, hepatocellular carcinoma (HCC) is the predominant form and is particularly prevalent worldwide [151]. Studies on Finnish and Japanese populations show an inverse correlation between serum sRAGE and CML-AGE levels and liver cancer risk [152,153]. Another study on Taiwanese individuals associated the *RAGE* gene polymorphism rs1800625 with early-stage HCC [154]. The expression of RAGE is higher in well-differentiated tumors among HCC patients and is associated with inflammation, carcinogenesis, and poor post-surgery outcomes, pointing to RAGE reduction as a possible therapeutic target [155]. In other liver diseases, a study on chronic hepatitis B patients found decreased serum sRAGE and tissue RAGE levels, correlated with hepatic necroinflammation [156]. Evidence suggests that AGEs influence liver cirrhosis by disrupting collagen architecture, with studies indicating elevated plasma AGE concentrations in cirrhosis patients, and increased serum CML serves as a marker for cirrhosis.

The liver plays a central role in clearing AGEs, primarily through hepatic sinusoidal endothelial cells and Kupffer cells, which handle 60% and 25% of this process, respectively [157]. Liver diseases impair this process, while AGE–RAGE interactions induce oxidative stress, affecting gene expression in hepatocytes, hepatic stellate cells, and Kupffer cells as well as activating pathways such as MAPK, PI3-K/AKT, and JAK2/STAT1 [158,159,160]. This leads to the release of profibrotic cytokines, transforming quiescent stellate cells into myofibroblasts, promoting fibrosis and cirrhosis [159]. The RAGE–ligand axis regulates MGO-mediated oxidative stress, contributing to hepatic steatosis, inflammation, fibrosis, and HCC [161]. Elevated levels of MGO-induced AGEs and reduced Glyoxalase-I in cirrhotic livers exacerbate liver injury and perpetuate the progression of cirrhosis [162]. Fatty acids stimulate CML accumulation, activating RAGE and increasing PAI-1, IL-8, and CRP production [163].

### 3.9. AGEs and Eye Diseases

AGEs accumulate in various regions of the eye, including the cornea, lens, retina, Bruch’s membrane, sclera, and optic nerve [164,165,166]. They are primarily associated with age-related or hyperglycemia-related macular degeneration, retinopathy, and cataracts [167,168,169]. For example, human lenses contain both crystallin monomers and polymers of argpyrimidine, with increased levels detected in cataractous lenses compared to noncataractous ones [170]. Moreover, accumulated argpyrimidine would induce apoptosis in lens epithelial cells [171]. High oxygen levels and oxidative stress in vascularized eye regions accelerate the formation of CML and other AGEs, making these areas particularly vulnerable [166]. Since retinal pigment epithelial cells and most lens cells lack proliferative capacity, AGE (such as CML and CEL)-induced damage is difficult to repair, leading to rapid accumulation [172]. Extracellular CML and MG-H1 alter the biochemical properties of ECM, contributing to retinal aging and age-related macular degeneration (AMD) [173]. Intracellular AGEs induce protein crosslinking, disrupting protein structure and function, which is cytotoxic and disrupts the inner blood-retinal barrier (iBRB) [5]. This upregulates VEGF and downregulates platelet-derived growth factor (PEDF), leading to oxidative stress, inflammation, and vascular dysfunction in the retina [174].

Elderly individuals with AMD have higher plasma levels of CML and pentosidine [175]. Elevated AGE levels have also been found in individuals with cataracts or retinopathy, both diabetic and non-diabetic [168,169]. In diabetic patients, glucosepane levels in the lens capsule are significantly higher, regardless of retinopathy status [165]. AGEs may also play a role in ocular surface diseases, such as diabetic keratopathy, by promoting inflammation and ROS pathways [176]. Additionally, serum high mobility group box-1 (HMGB1) levels are elevated in children with spring catarrhal conjunctivitis [177].

Intra-lenticular AGEs induce lens epithelial cell senescence, triggering a mesenchymal transition in adjacent cells, leading to posterior capsule opacification (PCO), a common complication after cataract surgery [178]. The interaction of AGEs with structural proteins such as myelin, tubulin, and lens proteins is linked to cataracts and other ocular disorders in aging and diabetes [179]. AGEs also affect collagen binding, reducing endothelial cell adhesion and causing vascular dysregulation, which is related to diabetic microvascular complications [180]. Accumulation of CML and CEL-modified albumin in retinal endothelial cells raises cell adhesion molecule levels, leading to capillary blockage and retinal ischemia [181]. Furthermore, the AGE–RAGE axis may contribute to diabetic retinopathy through the involvement of adiponectin [182].

### 3.10. AGEs and Lung Diseases

AGEs are increasingly recognized as key players in the pathogenesis of various lung diseases, including pulmonary fibrosis, acute lung injury (ALI), cancer, and infectious diseases. AGEs accumulate in lung tissues and interact with RAGE, triggering multiple signaling pathways that drive inflammation and fibrosis, ultimately leading to lung functional decline. Notably, RAGE is highly expressed in the lungs, particularly in alveolar epithelial cells, making its dysregulation a significant factor in the development of lung disorders [10].

In idiopathic pulmonary fibrosis (IPF) lungs, both pentosidine and CML were significantly increased [183]. Additionally, in the induced sputum of asthma patients, pentosidine levels were markedly increased, suggesting a potential role in impaired pulmonary function [184]. Furthermore, lower plasma levels of sRAGE have been associated with greater disease severity and poorer outcomes, suggesting that sRAGE plays a protective role by neutralizing the harmful effects of AGEs [185]. Moreover, studies using RAGE-deficient mice have shown that these animals are largely protected from bleomycin-induced lung fibrosis, further emphasizing the role of the AGE–RAGE axis in promoting fibrotic changes. The absence of RAGE in these mice leads to reduced pulmonary levels of pro-fibrotic cytokines such as TGF-β and PDGF, which are critical mediators of fibrosis [186]. Additionally, in cystic fibrosis-related diabetes (CFRD), elevated plasma levels of AGEs correlate with decreased lung function, indicating the potential for AGEs to exacerbate lung damage in this context [187]. Collectively, these studies suggest that the AGE–RAGE axis not only contributes to the progression of fibrosis but that sRAGE functions as a crucial protective factor, potentially offering a therapeutic target to mitigate the advancement of fibrosis in lung diseases.

RAGE is highly expressed in lung tissue under normal conditions, with its expression intensifying during inflammatory conditions such as ALI and acute respiratory distress syndrome (ARDS). Matrix metalloproteinase-9 (MMP-9) plays a role in ALI by influencing the release of sRAGE, and its knockdown exacerbates sepsis-induced ALI and inflammation by decreasing sRAGE levels. Conversely, administration of sRAGE mitigates inflammation and oxidative stress, improving overall outcomes [188]. In the context of infectious diseases like COVID-19, the AGE–RAGE axis has been implicated in the heightened morbidity and mortality observed in elderly patients and those with pre-existing conditions such as diabetes, obesity, and cardiovascular diseases, which are all associated with elevated AGE levels [189]. DM has been linked to a higher risk of severe complications and increased hospitalization, with pre-existing conditions in DM patients, such as endothelial dysfunction and a prothrombotic state, amplifying RAGE signaling, worsening lung inflammation, and thus increasing COVID-19 mortality [190]. Moreover, a significant association between COVID-19 severity and serum sRAGE levels has been established, with higher sRAGE levels observed in patients with severe COVID-19 compared to those with non-severe cases [191]. Blood samples from symptomatic COVID-19 patients revealed that elevated levels of RAGE and the viral antigens were strongly associated with the development of severe disease. The findings suggest that both biomarkers could help identify high-risk patients in emergency settings [192].

The AGE–RAGE axis plays a complex and multifaceted role in lung cancer progression, as CML has been shown to increase in both glycation and cancer models, suggesting a connection between glycation processes and cancer development [193]. While RAGE is highly expressed in normal lung tissue, its expression is often downregulated in cancerous tissues. This downregulation is associated with a significant reduction in sRAGE levels in the serum of lung cancer patients [194]. A study confirmed that serum sRAGE levels, as well as RAGE expression, are lower in lung cancer patients compared to healthy controls, with lower sRAGE levels correlating with increased lymph node involvement [195]. Concurrently, RAGE ligands such as HMGB1 and S100 proteins are upregulated in lung cancer tissue, contributing to the disease’s pathogenesis [194]. Additionally, the presence of RAGE gene polymorphisms has been linked to both the initiation and progression of lung cancer, indicating its potential as a diagnostic and prognostic biomarker [194].

The downregulation of RAGE in lung cancer cells reduces cell-to-cell and cell-to-substrate communication, thereby facilitating tumor progression [196]. Moreover, RAGE overexpression has been shown to inhibit cancer cell growth through p53-dependent mechanisms, although this overexpression also promotes tumor metastasis and the accumulation of tumor-associated macrophages via ERK signaling [197]. RAGE has also been implicated in the resistance of brain metastases to whole-brain radiotherapy through the S100A9-RAGE-NF-κB-JunB pathway, with targeting this pathway showing potential to improve treatment outcomes [198]. Overall, the intricate role of RAGE in lung cancer highlights its potential as both a biomarker and a therapeutic target in the treatment of lung cancer and its metastases.

### 3.11. AGEs and Obesity

Obesity, defined as excessive fat accumulation with a body mass index (BMI) of 30 or higher in adults, is not only a global epidemic but also a significant risk factor for various fetal diseases [199].

In recent years, the role of AGE accumulation in the development of obesity has garnered widespread attention. Early studies indicated that plasma AGE levels were lower in obese children [200]. A meta-analysis also observed an inverse association between circulating AGEs and BMI in adults [201]. However, circulating AGEs were reported to be elevated in unhealthy obese individuals at risk for metabolic syndrome [202]. The relationship between circulating AGE levels and obesity following in-body metabolism remains controversial. Notably, obese patients exhibited significant accumulation of AGEs, particularly CML, and higher RAGE expression in adipose tissue (AT), compared to non-obese subjects [203]. Due to the strong influence of serum CML concentrations on AT, CML might preferentially accumulate in AT [204], leading to lower circulating levels of CML in obese individuals [203]. Recent research revealed high AGE-to-sRAGE ratios in overweight and obese children [205]. This finding implies that monitoring the ratio of AGEs to sRAGEs could enhance our understanding of the mechanisms behind obesity and its associated complications, providing valuable insights for potential interventions.

Increasing research suggests that the AGE–RAGE axis is involved in AT dysfunction and the development of obesity. First of all, AGE formation impaired the proliferation and osteogenic differentiation potential of adipose-derived stem cells (ASCs) by suppressing the Wnt/β-catenin signaling pathway and increasing DNA methylation [206]. In contrast, in vitro studies have shown that exposure to AGEs inhibited the adipogenesis differentiation of human mesenchymal stem cells, with minimal impact on osteogenic development [207]. In addition to modulating the fate of ASCs, the AGE–RAGE axis has the potential to regulate lipolysis and adaptive thermogenesis by inhibiting the protein kinase A (PKA)-mediated phosphorylation of hormone-sensitive lipase (HSL) and the p38 MAPK pathway [208]. AGEs also suppressed apolipoprotein E expression in adipocytes, thereby reducing intracellular triglyceride synthesis both in vitro and in vivo [209]. Furthermore, the accumulation of AGEs in the ECM impaired glucose uptake and led to insulin resistance in human primary adipocytes by activating the AGE/RAGE/Diaphanous 1 axis [210,211]. AGE-stimulated adipocyte hypertrophy was accompanied by the downregulation of insulin-sensitive genes, such as glucose transporter type 4 (GLUT4) and adiponectin, which further impaired glucose uptake, insulin signaling and adipokine secretion [212].

### 3.12. AGEs and Intestinal Diseases

The gastrointestinal tract harbors trillions of microorganisms, collectively known as the gut microbiota, which play a critical role in maintaining host health [213]. Approximately 10% of dAGEs are absorbed by the small intestine and enter circulation, with some excreted in the urine [214]. The remaining AGEs, along with amino acids and peptides released during epithelial desquamation, are eliminated through feces [215]. These AGEs, such as CML, may be partially degraded and metabolized by the gut microbiota, serving as potential nutrients that influence host health by modulating inflammatory responses, metabolic processes, and immunoregulation [216]. This interaction can also affect the composition and function of the microbiome, thereby impacting overall health through changes to gut architecture [217].

Inflammatory bowel disease (IBD), encompassing conditions like ulcerative colitis and Crohn’s disease, is strongly linked to AGE/RAGE accumulation in the gastrointestinal tract [218]. Elevated pentose glycosides are observed in the gastrointestinal tissues of IBD patients, with urinary concentrations rising during active disease phases [219]. The high levels of AGE/RAGE in IBD may accelerate intestinal fibrosis, often associated with epithelial-mesenchymal transition [220]. Recent studies also suggest that AGE/RAGE signaling may serve as predictors for postoperative recurrence in Crohn’s disease [221,222]. Specifically, RAGE expression is significantly increased in the inflamed intestinal tissues of Crohn’s disease patients [223]. Dietary intake of CEL has been associated with an increased risk of Crohn’s disease [224]. Conversely, in ulcerative colitis or patients with penetrating behavior, sRAGE levels are reduced and inversely correlate with disease activity [225].

The interaction between RAGE polymorphisms, elevated RAGE levels, oxidative stress, and inflammation may contribute to the progression of IBD to colorectal cancer (CRC) [218]. A retrospective study of 133 colorectal adenocarcinoma cases found that AGE expression correlated with cancer histological grade, suggesting that AGEs may play a role in both early and late colorectal carcinogenesis [226]. Circulating glyceraldehyde-derived AGEs are significantly correlated with CRC-specific mortality [227]. Interestingly, pre-diagnosis sRAGE levels are negatively associated with CRC risk in men [228]. In women with a BMI of 25 or greater, those with the highest sRAGE levels exhibited a reduced risk of CRC [229]. However, other studies have found a positive correlation between sRAGE and CRC mortality, highlighting the complex role of RAGE in cancer outcomes [230]. In advanced CRC tumors, glyoxalase 1 (GLO1) levels are depleted, while methylglyoxal remains elevated, further implicating AGEs in cancer progression [231].

Dietary interventions have shown that AGE restriction can alter the gut microbiota. A study of peritoneal dialysis patients revealed that one month of AGE restriction (measured by CML and MG levels) led to a significant reduction in the abundance of *Prevotella copri* and *Bifidobacterium animalis* and an increase in *Clostridium citroniae*, *Clostridium hathewayi*, *Alistipes indistinctus*, and *Ruminococcus gauvreauii* [232]. This microbiota shift may contribute to cardiovascular events. Similarly, a Dutch trial with 718 participants found that higher dietary AGE (quantified by CML, CEL, and MG-H1 levels) intake was linked to a lower abundance of *Barnesiella*, *Colidextribacter*, and *Terrisporobacter*, while increasing *Coprococcus*, *Dorea*, and *Blautia* [214]. Studies on adolescents suggest that Maillard reaction products reduce *lactobacilli* and *bifidobacterial* levels, further supporting the notion that AGEs can significantly reshape the microbiota [233].

AGEs have been associated with a reduction in the abundance of beneficial microbes that produce short-chain fatty acids (SCFAs), potentially contributing to insulin resistance [234]. However, some glycated products also serve as substrates for SCFA production [235]. AGEs may disrupt intestinal homeostasis by interacting with intestinal glial cells, promoting inflammation and oxidative stress [236]. They also upregulate SP1 expression, potentially promoting invasion and metastasis in colorectal cancer through the RAGE/ERK/MMP2 signaling pathway [237]. Moreover, methylglyoxal-derived AGEs can compromise the integrity of the intestinal epithelial cell barrier, increasing permeability, triggering inflammatory responses, and further exacerbating oxidative stress, all of which contribute to gastrointestinal disorders and cancer progression [238].

### 3.13. AGEs and Reproductive Diseases

AGEs are also strongly linked to reproductive disorders, a connection that is often overlooked. The involvement of AGEs in ovarian dysfunction was demonstrated in the study of polycystic ovary syndrome (PCOS) [239,240]. Elevated AGE levels could be detected in both the serum and the ovary of PCOS patients. Further evidence from the human granulosa cell line model (KGN) showed that the AGEs in vitro could interfere with LH action and lead to sustained abnormal activation of the ERK1/2 pathway, which leads to impaired follicular development and hence ovulatory dysfunction associated with PCOS. Another experiment on the culture of the KGN cells with insulin or human glycated albumin (HGA; rich with AGEs), alone or in combination, demonstrated that AGEs within the ovary alter glucose metabolism and folliculogenesis [241]. Besides PCOS, AGEs may also contribute to the formation of endometriosis [242], although the mechanisms are not well understood. For reproductive dysfunction during aging, the AGE-RAGE-VEGF signaling may lead to reproductive environment changes and affect reproductive function [243]. More directly, the relationship between AGEs and infertility has been documented in several studies, suggesting the important role of AGE accumulation in ovarian dysfunction and poorer outcomes in women with elevated AGEs and undergoing in vitro fertilization (IVF) [243]. As reported, the toxic AGEs (TAGEs) pentosidine and CML accumulate in the follicular fluid, and the TAGE level in serum is negatively correlated with follicular growth, fertilization, and embryonic development. Lower concentrations of pentosidine in the follicular fluid and TAGEs in the serum were even taken as the most significant predictors for achieving pregnancy in those patients undergoing IVF [244].

The deleterious effects of AGEs on the ovary have also been evidenced in animal experiments. Female mice subjected to a diet high in AGEs had longer diestrus phases, significant alterations in genes involved in steroidogenesis and folliculogenesis, and fewer corpora lutea in their ovaries, suggesting the dysfunction of the ovary [245].

As they accumulate in female ovaries, AGEs can also deposit in the male testes, epididymis, and sperm, especially in men with diabetes [246], and possibly play a role in diabetes-related infertility [13]. Levels of AGEs were significantly increased in the spermatozoa of diabetic men, and RAGE expression was also elevated in both spermatozoa and seminal plasma [247,248]. However, the direct clinical evidence of AGE-induced infertility is limited. In animal studies, an AGE-rich diet induced histopathological damage in the testes and epididymides of mice, accompanied by a decrease in normal sperm morphology, epididymal sperm reserve [249], and sperm function [250]. Oxidative stress is one of the key factors in AGEs involved in male infertility [251]. As reported, AGEs can inhibit testosterone production and secretion in rat Leydig cells by inducing oxidative stress and endoplasmic reticulum stress [252].

## 4. AGEs and Interventions

### 4.1. AGE Inhibitors

Compounds that inhibit AGEs hold potential as therapeutic approaches for treating various AGE-related diseases. They act through multiple mechanisms, including preventing the interaction between reducing sugars and amino acids in early glycation, scavenging free radicals, sequestering Amadori intermediates to block late glycation stages, and breaking AGE crosslinks [253,254]. These AGE inhibitors can be categorized into two main groups based on the sources: naturally occurring polyphenolic compounds and synthetic small molecules [253].

Currently, most research is conducted at the in vitro and animal levels, with only a limited number of compounds undergoing clinical studies. Additionally, some drugs (such as aspirin and diclofenac) are widely used in clinical practice. However, there are no specific clinical studies focused on inhibiting AGEs to improve associated diseases, therefore, their clinical dosages have not been established. The following table outlines the mechanisms, indications, and clinical dosages (where available) of synthetic compounds (Table 1). Clinical studies on these inhibitors have primarily focused on diabetes and its complications, as well as related metabolic disorders. However, whether these small molecules can be applied to other AGE-related diseases (skin, joints, gut, and aging) requires further evidence.

Several widely used medications, including metformin, benfotiamine, and pyridoxamine, have also been shown to inhibit AGE formation, presenting potential new therapeutic applications. Despite over two decades of research into AGE inhibitors, clinical trials involving compounds like aminoguanidine and pyridoxamine have shown significant side effects [1,255]. To date, the FDA has not approved any treatments specifically for AGE-related diseases. However, recent advances in newly synthesized small molecules with potent AGE-inhibitory effects offer promising prospects for future therapies.

**Table 1:** The mechanisms and indications of synthetic compounds as AGE inhibitors.

Structure	Name	Mechanisms	Potential Indications	Clinical Dosage	References
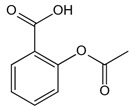	Aspirin	Inhibits the glycation process via acetylating free amino groups of proteins, thereby blocking the attachment of reducing sugars	Diabetes and its late-stage complications	/	[256,257,258]
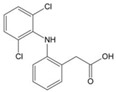	Diclofenac	Protects proteins from sugar attachment due to its non-covalent interaction with proteins (such as serum albumin)	Anti-inflammation	/	[259]
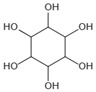	Inositol	Scavenging of glucose	Diabetes, cataracts, and diabetic retinopathy	/	[260,261,262]
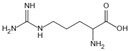	Arginine/lysine	Protein-glycation inhibitor, competitive attachment with glucose	Cataracts	/	[263,264]
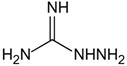	Aminoguanidine	Carbonyl scavenger, reacts with β-dicarbonyl intermediates induced by glycation	Diabetic complication	/	[265,266,267]
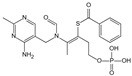	Benfotiamine	Accelerates the precursors of AGEs toward the pentose phosphate pathway	Alzheimer’s disease	600 mg/d	[268,269,270]
			type 2 diabetes	900 mg/d	
			diabetic neuropathy	150–320 mg/d	
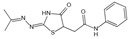	2- Isopropylidenehydrazono-4-oxo-thiazolidine-5-ylacetanilide (OPB-9195)	Carbonyl scavenger, metal-ion chelation	Diabetic complication	/	[271]
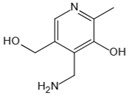	Pyridoxamine	Mental-ion chelation, radical scavenging properties, sequestering the ROS and RNS	Diabetic retinopathy	200 mg/d	[272,273,274,275,276]
			Atherosclerosis	1200–2400 mg/d	
			Diabetic nephropathy	100–500 mg/d	
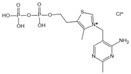	Thiamine pyrophosphate	Dicarbonyl scavenger	Vascular complications of diabetes	/	[277,278]
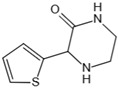	Tenilsetam	Dicarbonyl scavenger, restrains the polymerization of lysozyme with 3-DG, transition metal ion chelator	Age-related neurodegenerative diseases	/	[265,279]
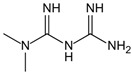	Metformin	Protects proteins against glycation and cross-linking, captures discarbonyls produced	Type 2 diabetes	1500–2550 mg/d	[280,281,282]
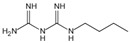	Buformin	Protects proteins against glycation and cross-linking, traps carbonyls of ammonia and MGO	Type 2 diabetes	/	[281]
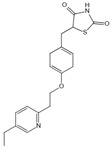	Pioglitazone	Carbonyl scavenger	Type 2 diabetes	30 mg/d	[283,284]
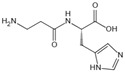	Carnosine	Reacts with sugars to prevent the formation of AGEs, protects proteins against glycation and cross-linking, and scavenges ROS	Vascular complications of diabetes	/	[285,286,287,288]
			Type 2 diabetes and diabetic nephropathy	1000 mg/d	
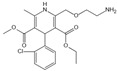	Amlodipine	Radical scavenging properties	Atherosclerotic lesions	/	[289,290,291]
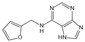	Kinetin	Radical scavenging properties	Alzheimer’s disease	/	[292,293]
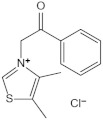	Alagebrium chloride (ALT-711)	AGE breaker, breaks down established AGE-related protein cross-link	Arteriosclerosis, hypertension, diastolic heart failure	420 mg/d	[294,295,296,297]
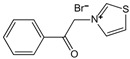	N-phenacylthiazolium bromide (PTB)	AGE breaker, cleaving α-diketone structure	Diabetic vascular complications, diabetic periodontitis	/	[298,299,300]
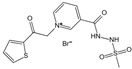	3-[[2-(Methylsulfonyl)hydrazinyl]carbonyl]-1-[2-oxo-2-(2-thienyl)ethyl]pyridinium bromide (TRC-4149)	AGE breaker, free radical scavenging activity	Diabetic vascular complications	/	[301,302,303]

### 4.2. Natural Products Inhibiting AGE Formation

Chemical compounds extracted from plants or animals, known as ‘natural compounds’, have exhibited unique pharmacological effects. Currently, natural compounds from plants and foods have shown excellent inhibitory activity against the formation of AGEs, with minimal toxicity [304]. Therefore, the screening of natural compounds that can be used as glycation inhibitors has been of great interest. This paper presents natural compounds with the potential to inhibit the formation of AGEs, categorized into six groups based on their structural properties: polyphenols, polysaccharides, terpenoids, vitamins, alkaloids, and peptides.

#### 4.2.1. Polyphenols

Polyphenols are classified into flavonoids, phenolic acids, stilbenes, and lignans based on the number of phenolic rings and the structural elements that connect them [305]. This paper will focus on flavonoids that can suppress the formation and accumulation of AGEs.

##### Flavonoids

Flavonols: Quercetin, as a flavonol glycoside, has been found to reduce plasma MGO concentrations in healthy (prehypertensive) adults, indicating its therapeutic potential for MGO-mediated diseases [306]. Quercetin could inhibit AGE formation by scavenging MGO and GO in vitro [307]. Another flavonol glycoside, rutin, could not only scavenge free radicals but also inhibit the formation of AGEs by chelating metal ions to form complexes [308]. Studies on rutin supplementation have demonstrated its ability to improve metabolic parameters and oxidative stress factors in T2DM patients, confirming the in vivo antiglycation capacity of rutin [309]. Other flavonols, such as kaempferol and myricetin, also play a role in preventing the formation and accumulation of AGEs through various mechanisms and have shown beneficial effects on antioxidant capacity in vivo [310].

Flavanonols: As one of the active ingredients in silymarin, taxifolin has been shown to trap endogenous MGO by forming mono-MGO adducts in diabetic mice, highlighting its potential for treating chronic diseases associated with AGEs [311]. The derivative of aromadendrin, aromadendrin-8-C-glucopyranoside, was found to inhibit the formation of AGEs in a dose-dependent manner [312], warranting further in-depth investigation.

Flavones: Luteolin, apigenin, and diosmin have been reported to inhibit AGE formation through various mechanisms, including the trapping of active dicarbonyl intermediates and the inhibition of ROS generation [310].

Flavanones: Naringenin and eriodictyol have the potential to suppress the formation of AGEs and mitigate AGE-induced oxidative stress and inflammation [313,314]. The enhanced inhibitory activity of eriodictyol, compared to naringenin, is attributed to the presence of an additional hydroxyl group in the B ring of eriodictyol [314].

Flavanols: Significant inhibition of AGE formation and accumulation through the trapping of MGO by (+)-catechin, (−)-epicatechin, and their derivatives, (−)-epicatechin-gallate and (−)-epigallocatechin-3-gallate (EGCG), has been demonstrated both in vitro and in vivo [310].

Chalcones: Curcumin has been suggested to inhibit the formation of AGEs by capturing MGO and preventing collagen cross-linking [315]. Apple polyphenols, including phloretin and its derivative phloridzin, could effectively trap dicarbonyls produced by late-stage glycosylation end products [316,317], potentially mitigating secondary complications associated with diabetes [318].

Isoflavonoids and Biflavonoids: One of the isoflavonoids, genistein, has been reported to be a potent aldose reductase inhibitor that traps MGO to form mono- and dual-MGO adducts in vitro, thereby reducing the formation of AGEs [319]. In addition, amentoflavone, which belongs to the class of biflavonoids, exerts its anti-AGE activity by scavenging free radicals, chelating divalent metal ions, and trapping dicarbonyl groups [307].

##### Phenolic Acids, Stilbenes, and Lignans

Phenolic acids are common plant secondary products with higher concentrations than polyphenolic flavonoids and are mainly divided into two groups: hydroxybenzoic acids and hydroxycinnamic acids [320]. An example of hydroxybenzoic acids is 7-O-galloyl-D-sedoheptulose, which reduced levels of AGEs and ROS in diabetic mice, demonstrating a protective effect in the early stages of diabetic kidney disease [321]. Among the well-studied hydroxycinnamic acids, caffeic acid, ellagic acid [322], and ferulic acid [323] have been shown to reduce the AGE formation and accumulation in diabetic rats. Rosmarinic acid could inhibit AGE-mediated crosslinking and alleviate liver fibrosis progression in mice [324]. These studies suggest that phenolic acids are potential anti-glycation compounds.

Resveratrol, a kind of stilbene, has been shown to inhibit the carbohydrate enzymes α-glucosidase and α-amylase while also reducing the formation of AGEs [325]. Resveratrol supplementation reduced glycated hemoglobin and had a protective effect in patients with T2DM [326]. Lignans extracted from *Cortex Eucommiae* protected against AGE-induced endothelial cell dysfunction in vivo and in vitro by modulating Nrf2/HO-1 signaling [327].

##### Anthocyanins

The anthocyanins from fruits and vegetables have been mostly reported to have antiglycative activities. Blueberry (*Vaccinium corymbosum*) anthocyanins extract (BAE) exhibits strong anti-glycation activity by scavenging glycosylated intermediates, like Schiff base, fructosamine, and α-dicarbonyl compounds, attenuating the molecular aggregation and amyloid-like fibril formation, preventing conformational modification, and inhibiting AGE-stimulated inflammation in RAW264.7 cells [328]. Similar activity was reported in other berries, like Chinese bayberry (*Myrica rubra*) [329], cranberry (*Vaccinium Oxycoccos*), strawberry (*Fragaria ananassa Duch*.), raspberry (*Rubus corchorifolius*), blackberry (*Rubus grandifolius Lowe*) [330], blackcurrant (*Ribes nigrum* L.) [331], sea buckthorn (*Hippophae rhamnoides*) [332], black chokeberry (*Aronia melanocarpa*) [333], goji berry (*Lycium barbarum* L.), and grape [334], which are all rich in anthocyanins. Bilberry (*Vaccinium myrtillus* L.) also contains vast amounts of anthocyanins and is reported to have anti-diabetic activities [335]. It has long been used for the prevention and treatment of diabetes [336]. In China, the drug Difrarel, which contains bilberry extracts as its primary ingredient, was approved for the treatment of diabetic retinopathy. In a clinical study of seventy-four healthy subjects, plasma protein-bound AGEs can be reduced partly by 3-month *Vaccinium myrtillus* extract supplementation [337]. In another more in-depth animal experiment, the rats were fed an AGE-rich diet with or without bilberry extracts for 80 weeks. The results showed that exposure to the AGE-rich diet led to varying degrees of accumulation of AGEs (free-CML, free-CEL, bound-CML, and bound-CEL) in different tissues. Notably, the addition of bilberry extracts significantly reduced AGE accumulation, especially in the kidney, skin, and brain. These results suggested that Vaccinium Myrtillus could be a promising natural product for the prevention of AGE accumulation [19]. In addition to berries, other plants such as purple corn, red rice, and *Clitoria ternatea* L. flowers also demonstrate anti-glycation activity due to their rich anthocyanin content.

The commonly consumed berry fruits had excellent MGO-trapping and antiglycative activities, which are positively associated with their total phenolic contents, as exhibited by some ex vivo experiments [331]. Moreover, certain anthocyanins from these anti-glycation plant extracts exhibit strong efficacy and unique mechanisms. Cyanidin-3-rutinoside can attenuate MGO-induced protein glycation and DNA damage via carbonyl trapping ability and scavenging ROS [338]. Cyanidin-3-O-glucoside exhibits masking-like function, carbonyl scavenging, and chemical chaperone activity in the inhibition of early and advanced glycation [339].

#### 4.2.2. Terpenes

Terpenoids, which are derivatives of terpenes, are widely distributed among various plant species and exhibit antioxidant and anti-glycation bioactivities [304]. Among the most extensively studied pentacyclic triterpenoids, oleanolic acid and ursolic acid have shown promise for clinical applications in the prevention or mitigation of glycation-related diseases [340]. Research indicates that ursolic acid may inhibit the activities of aldose reductase and sorbitol dehydrogenase, resulting in a reduction in hyperglycemia and hepatic glucose production in diabetic mice [341]. Aucubin, which belongs to another class of terpenoids known as iridoids, has been shown to directly trap MGO in vitro and prevent the formation and accumulation of AGEs in vivo, indicating its potential for treating AGE-related chronic diseases [342].

#### 4.2.3. Polysaccharides

Polysaccharides are macromolecules composed of at least ten monosaccharides that are polymerized into straight or branched chains via glycosidic bonds, exhibiting antioxidant and hypoglycemic properties [343]. Astragalus polysaccharides have been shown to decrease the formation of AGEs by protecting insulin activity, stimulating insulin secretion, and improving glucose and lipid metabolism [344]. Recently, the degraded polysaccharides from *Auricularia auricula-judae* have been shown to inhibit the formation of AGEs in a dose-dependent manner [345]. Due to the relatively low toxicity of most polysaccharides extracted from plants, they are expected to be a promising source of glycosylation inhibitors. However, further research is required to delineate the molecular mechanisms involved.

#### 4.2.4. Alkaloids

As a class of nitrogenous organic compounds, alkaloids exhibit a diverse range of pharmacological and biological activities due to their various structures. One of the most extensively studied alkaloids, berberine, has been shown to increase the production of nitrogen oxides and promote endothelium-dependent vasodilation [346]. Additionally, it exerted protective effects against endothelial dysfunction induced by high glucose levels in vitro [346]. Berberine can reduce blood glucose levels and inhibit the accumulation of AGEs in diabetic rats [347]. A meta-analysis revealed that berberine exerted a comparable therapeutic effect on T2DM without serious side effects [348], suggesting that berberine has a promising clinical application in anti-glycation.

#### 4.2.5. Vitamins

Vitamin C is a kind of water-soluble vitamin that competes with glucose in binding to proteins, thereby inhibiting the production of AGEs in vitro [349]. In addition to inhibiting the sorbitol pathway, vitamin C also reduces fructose production in diabetic patients [349]. Vitamin E, a lipid-soluble vitamin, has been shown to suppress hemoglobin glycosylation and the formation of AGEs [350]. Pyridoxamine, a member of the vitamin B6 family, has demonstrated the ability to scavenge free radicals and chelate metal ions, making it a commonly used glycation inhibitor in clinical settings [351].

#### 4.2.6. Peptides

Carnosine, a natural dipeptide synthesized in the body, was found in high concentrations in brain and muscle tissues, with levels ranging from approximately 1 to 20 mmol/kg [352]. Carnosine exhibited antioxidant and anti-glycation properties, effectively preventing oxidative stress and the formation of AGEs in D-gal-induced aging rats [353]. In addition, research indicates that glutathione is more effective than carnosine in preventing glucose glycation [354].

### 4.3. Health Interventions for AGEs

Various environmental factors, such as high-carbohydrate and high-calorie diets, high-temperature cooking, cigarette smoking, and a sedentary lifestyle, may contribute to the formation of AGEs [4]. To summarize, AGEs and metabolic disorders are established on the concept of the ‘common soil’ [14]. Although ultraviolet rays, ionizing radiation, and air pollution are unavoidable, preventive action against glycation by lifestyle adjustment may be warranted [4]. For a healthier diet, it is advisable to limit highly processed food and instead choose options rich in whole grains, dairy products, nuts, and legumes [355,356], prioritizing foods with a low glycemic index and low fat content, along with a variety of natural fresh ingredients [357]. Moreover, adopting a Mediterranean diet, which is rich in monounsaturated fatty acids and minimally processed natural foods, can help reduce AGE levels by mitigating postprandial oxidative stress and inflammation [358]. Cooking strategies to minimize exposure to food AGEs include avoiding the use of repeatedly heated oils, opting for lower cooking temperatures when possible, and incorporating acidic ingredients like vinegar or lemon juice to lower AGE formation [359]. Additionally, it is advisable to choose cooking methods such as steaming, stewing, and boiling rather than frying or grilling [360]. In addition to dietary restrictions, enhancing physical activity and quitting smoking are also crucial interventions and practical strategies.

## 5. Discussion

Over the past few decades, AGEs have garnered widespread attention as crucial factors associated with aging and various chronic diseases. AGEs are a class of compounds formed through non-enzymatic glycation (i.e., the Maillard reaction), and their progressive accumulation in tissues and organs can lead to cellular damage, disrupt normal physiological functions, and contribute to the onset of multiple diseases, including diabetes, cardiovascular diseases, neurodegenerative diseases, chronic kidney disease, skin aging, metabolic disorders, reproductive disorders, and degenerative bone diseases.

Traditionally, AGE levels have been most closely linked to diabetes and its complications, such as CVDs and CKD, as their severity is positively correlated with AGE accumulation. This is primarily because the persistent hyperglycemic environment in diabetic patients naturally facilitates AGE formation. However, as research on AGEs has deepened, new theories have emerged, suggesting that AGEs are not merely byproducts of diabetes but may also play a causal role in its development. Additionally, many prediabetic individuals exhibit significantly elevated AGE levels, which may be closely related to certain complications that appear even before a formal diabetes diagnosis.

Furthermore, AGE-induced skin aging has become a significant health concern in recent years. The cross-linking of collagen and elastin caused by AGE leads to a decline in skin elasticity, while AGE-induced melanin deposition results in a dull complexion.

Given these findings, studying the formation mechanisms and pathogenic effects of AGEs is of great significance for understanding aging and chronic diseases, particularly the onset and progression of diabetes and its related syndromes.

The formation of AGEs involves multiple chemical processes. Changes in metabolic levels, along with lifestyle factors such as hyperglycemia, high-calorie diets, smoking, and a sedentary lifestyle, can accelerate the synthesis and accumulation of AGEs. AGEs primarily exert their pathological effects by trapping and crosslinking the protein or by interacting with their receptor RAGE, triggering oxidative stress, inflammatory responses, and apoptosis, which further exacerbate tissue and organ damage. As a result, reducing the formation and accumulation of AGEs has become a key focus in current biomedical research.

Currently, interventions targeting AGEs mainly fall into two categories: chemically synthesized small-molecule inhibitors and natural compounds. Chemically synthesized small-molecule inhibitors can reduce the harmful effects of AGEs by interfering with their formation pathways or breaking down already-formed AGEs. However, due to the potential side effects associated with some small-molecule inhibitors, their clinical applications have been limited. As a result, researchers have increasingly focused on bioactive compounds derived from natural foods and plants in recent years.

Natural compounds, particularly polyphenolic compounds from plants, such as anthocyanins, quercetin, and catechins, have been proven to possess strong anti-glycation properties. These compounds could inhibit the formation of AGEs through mechanisms such as lowering the blood glucose level, trapping reactive carbonyl compounds, inhibiting oxidative stress, and blocking protein glycation sites. At the same time, they may also promote the degradation of AGEs and protect tissues from AGE-induced pathological damage.

To date, many natural compounds or plant extracts have been clinically tested for their ability to reduce AGEs and protect against AGE-related damage. Three months of intervention with *Vaccinium myrtillus* extract (600 mg/day) in healthy subjects reduced the CML level, though the CEL level was not significantly changed [337]. Two months of resveratrol supplementation (1000 mg/day) can effectively reduce Hb1Ac levels in the blood of T1D patients and exert anti-diabetic effects [361]. Supplementation of vitamin B (133 mg/day), C (500 mg/day), and E (400 mg/day) in combination with anti-diabetic drugs can improve the rate of development of retinopathy in patients with T2DM and reduce circulating AGEs [362]. Many plant extracts, such as *Moringa oleifera* leaf (2400 mg/day) and green tea extract (800 mg/day), also reduce glycemic outcomes [363,364]. Currently, the combination of small-molecule inhibitors and herbal medicines is also being used in the treatment of diabetes, which is expected to be an effective option for lowering blood glucose in the clinic [365,366]. At the same time, numerous animal studies have also demonstrated the effectiveness of these natural compounds in reducing AGE levels and protecting against related dysfunction.

Although natural compounds exhibit great potential in inhibiting AGE formation, their clinical application remains somewhat limited due to their low bioavailability. For instance, polyphenolic compounds are often easily degraded in the digestive tract, leading to low absorption efficiency in the body. Therefore, future research should focus on improving the bioavailability of these compounds by enhancing their stability and absorption efficiency through techniques such as nanocarriers, liposomal encapsulation, or formulation optimization.

Additionally, to further advance the application of natural anti-AGE compounds, a thorough evaluation of their safety and efficacy is necessary. While some natural compounds have shown promising effects in animal studies and clinical research, more randomized controlled trials (RCTs) are needed to verify their long-term safety and therapeutic effectiveness.

From the perspective of developing drugs to reduce AGEs, we still have a long way to go. However, some natural products that serve both as food and medicine have a long history of consumption, demonstrating good safety profiles while also exhibiting strong AGE-lowering activity. These natural products, such as bilberry extract, green tea extract, and resveratrol, can be prioritized as nutritional interventions to help protect against the health risks associated with AGEs.
